# Development and Validation of a Prognostic Score to Predict Survival in Adult Patients With Solid Tumors and Bone Marrow Metastases

**DOI:** 10.1097/MD.0000000000000966

**Published:** 2015-06-12

**Authors:** Wen-Chi Chou, Kun-Yun Yeh, Meng-Ting Peng, Jen-Shi Chen, Hung-Ming Wang, Yung-Chang Lin, Chien-Ting Liu, Pei-Hung Chang, Cheng-Hsu Wang, Ping-Tsung Chen, Yu-Shin Hung, Chang-Hsien Lu

**Affiliations:** From the Division of Hematology and Oncology, Department of Internal Medicine, Chang Gung Memorial Hospital at Linkou and Chang Gung University School of Medicine (W-CC, M-TP, J-SC, H-MW, Y-CL, Y-SH); Graduate Institute of Clinical Medical Sciences, College of Medicine, Chang Gung University, Taoyuan (W-CC, C-HL); Division of Hematology and Oncology, Department of Internal Medicine, Chang Gung Memorial Hospital at Keelung, Keelung (K-YY, P-HC, C-HW); Division of Hematology and Oncology, Department of Internal Medicine, Chang Gung Memorial Hospital at Kaohsiung, Kaohsiung (C-TL); Division of Hematology and Oncology, Department of Internal Medicine, Chang Gung Memorial Hospital at Chiayi, Chiayi (P-TC, C-HL), Taiwan.

## Abstract

Bone marrow metastasis (BMM) in patients with solid cancers is indicative of advanced-stage disease with a poor prognosis. The clinical features and outcomes remain unclear. We aimed to develop a scoring system to predict survival in these patients to help with clinical decision making.

A total of 165 adult patients diagnosed with solid cancers and BMM between 2000 and 2014 were selected as the derivation cohort. A risk model was developed using multivariate logistic regression from the derivation cohort and a marrow metastases prognostic score (MMPS) was generated. An independent cohort of 156 patients from 3 other hospitals was selected using the same recruiting criteria to validate the MMPS as a predictor of survival.

The MMPS was calculated based on 4 independent prognostic variables: the Eastern Cooperative Oncology Group performance scale, site of cancer, platelet count, and neutrophil-to-lymphocyte ratio. Patients in both the derivation and validation cohorts were stratified into good, intermediate, and poor prognostic groups based on their MMPS. The median survival in each risk group of the derivation cohort was 241, 58, and 11 days for the good, intermediate, and poor prognostic groups, respectively, and 305, 65, and 9 days, respectively, in the validation cohort. The c-statistic values for prediction of mortality at 3, 6, and 12 months were significantly higher for the MMPS than for the Eastern Cooperative Oncology Group performance scale in both cohorts.

We developed a risk model that accurately predicted survival in adult patients with solid cancers and BMM. This scoring system may help patients and clinicians with treatment decisions.

## Abstract 2

Supplemental Digital Content is available in the text

## INTRODUCTION

Bone marrow metastasis (BMM) from solid cancers is uncommon. Previous retrospective studies have indicated that the incidence ranges from 0.17% to 1.19% in adult patients with solid tumors.^1,2^ Solid cancers that present with BMM in adults are likely to arise from the breast, prostate, lung, or stomach.^1–9^ However, all solid tumors have the potential to metastasize to the bone marrow.^4–6,10–14^

Cancer patients who present with leukoerythroblastosis and unexplained anemia and/or thrombocytopenia should be evaluated for BMM.^3–9,15^ A bone marrow biopsy is the standard procedure used to make a definitive diagnosis^3,16,17^ and to exclude both nonmalignant causes and primary hematologic disorders. Because the procedure is invasive, it is not typically performed in patients with comorbidities and poor prognoses. Thus, the incidence of BMM from solid cancers is likely underestimated.

Metastasis to the bone marrow is usually indicative of disseminated cancer, a rapidly deteriorating clinical course, and a dismal prognosis. In general, the median survival is of the order of weeks.^2,4–8^ Previous studies have aimed to identify prognostic factors in patients with solid tumors and BMM. Primary tumors of the prostate or breast,^1,6^ good performance status,^6^ lack of thrombocytopenia,^6^ and antitumor therapy^5–7^ were the most frequently reported positive prognostic factors for patients with BMM. Unfortunately, limitations in past studies have led to inconsistent results. First, the data were collected from single institutes and involved small patient cohorts.^1–8^ Second, several studies reported prognostic factors in patients with BMM that were cancer type specific.^1,7,8^ Therefore, the results were not generalizable. Finally, antitumor therapy should not have been classified as a prognostic factor because patient performance status, cancer type, and adequacy of the bone marrow reserve confounded this factor.

We recently found that performance status, primary tumor site, platelet count, and antitumor therapy were significant prognostic factors among 83 adult patients with solid cancers and BMM.^6^ We expanded our patient numbers and collected data for the survival analysis using our institutional cancer center registry. The aim of this study was to develop and validate a prognostic score for predicting the outcomes of patients with solid cancers and BMM.

## PATIENTS AND METHODS

### Patient Selection

A retrospective study was conducted on a derivation cohort of patients with solid cancers and BMM between January 2000 and June 2014 at the Chang Gung Memorial Hospital (CGMH) Linkou Medical Center in northern Taiwan. Patients that were >20 years old, received a bone marrow biopsy because of an abnormal peripheral complete blood count (CBC), and were diagnosed with BMM by bone marrow examination were included. Patients with hematologic malignancies involving the bone marrow, those with an inconclusive diagnosis of BMM after a bone marrow biopsy, and those lost to follow-up after the biopsy were excluded. Patients with incomplete CBC data within 7 days before a bone marrow biopsy were also excluded. An independent cohort of consecutive patients was selected using the same criteria from 3 hospitals affiliated with the CGMH (The Keelung, Chiayi, and Kaohsiung branches of the CGMH) was selected for validation. The institutional review board for all branches of the CGMH approved this study on October 23, 2014 (103–4570B).

### Data Collection

We collected data on patient demographics, primary tumor site, histological type, Eastern Cooperative Oncology Group performance scale (ECOG scale), predominant CBC abnormality that indicated a bone marrow biopsy should be performed, whether the cancer was initially diagnosed because the patient presented with BMM, the CBC findings at the time of BMM diagnosis, the use of systemic antitumor therapy after BMM, and survival times. If BMM was diagnosed before or within 7 days of the primary cancer diagnosis, it was classified as BMM present at the initial diagnosis. The neutrophil-to-lymphocyte ratio (NLR) was calculated by dividing the blood neutrophil count by the blood lymphocyte count. Cytotoxic chemotherapy, targeted therapy, and hormone therapy specific for prostate or breast cancer were considered systemic antitumor therapies. The interval between the primary cancer diagnosis and BMM was calculated from the date of the primary cancer diagnosis to the date of bone marrow examination. Survival times were calculated from the date of the bone marrow examination to the date of death. The dates of the primary cancer diagnosis, diagnosis of BMM, and death of each patient were obtained from either the institutional cancer center registry or the National Register of Death Database in Taiwan. Follow-up of all patients was conducted until death or until the end of the study (December 31, 2014).

### Statistical Analysis

The basic patient demographic data were summarized as n (%) for categorical variables and mean with range, standard error (SE), or 95% confidence interval (CI) for continuous variables. Fifteen predefined variables (Supplementary appendix, http://links.lww.com/MD/A302), which were recorded at the time of the bone marrow biopsy, were then evaluated in the derivation cohort to ascertain the impact of each variable on patient survival. These key, potential prognostic variables were selected because the data were nearly complete and could be widely representative, making the findings clinically applicable. An a priori statistical analysis plan was approved. Seven of 15 variables, including sex, ECOG scale, primary tumor site, histological differentiation, present with extra bone and BMM, platelet count and NLR, with *P* values of <0.10 in a univariate analysis were analyzed using a multivariate model. A multivariate, Cox proportional hazard model with backward selection was performed to determine which factors were independently predictive of survival. Fractional polynomials were used for continuous variables.^18^ A risk model was developed based on the multivariate logistic regression analysis. The β-coefficients from the risk model were used to determine the marrow metastases prognostic score (MMPS) for calculating survival time. Receiver operating characteristic (ROC) curves and the area under the curve (c-statistic) for the outcome of mortality at 3, 6, and 12 months were calculated to determine the accuracy of the MMPS. Patients were stratified into 3 prognostic groups according to the total MMPS. The MMPS was then validated by calculating the survival time and generating a c-statistic value for mortality at 3, 6, and 12 months in the validation cohort.

Overall survival in the prognostic categories was calculated according to the Kaplan-Meier method. Log-rank tests were used to determine significant differences between survival curves. Differences in the c-statistics between the MMPS and the ECOG performance scale were calculated using the MedCalc software, Version 12.7.1.0 (MedCalc, Ostend, Belgium). Additional statistical analyses were performed with the SPSS 17.0 software (SPSS Inc, Chicago, IL). All statistical assessments were 2-sided. A *P* value of <0.05 was considered significant.

## RESULTS

### Patient Characteristics

**FIGURE 1:**
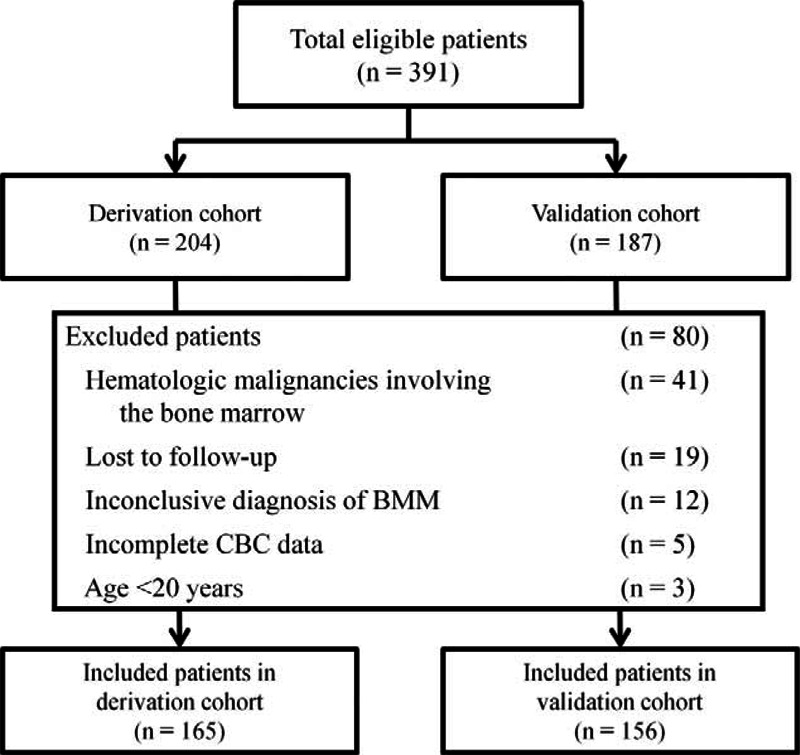
CONSORT diagram. BMM = bone marrow metastases, CBC = complete blood count.

A total of 165 and 156 patients with solid tumors and BMM were included in the derivation and validation cohorts, respectively (Figure 1). The demographic and clinical characteristics of the patients are shown in Table 1. Similar distributions in age, ECOG performance scale, interval between the time of primary tumor diagnosis and BMM, the main CBC abnormality indicating bone marrow biopsy, and organs involved in metastasis were observed in the derivation and validation cohorts. Patients in the validation cohort were more likely to be male, initially present with BMM, have a nonadenomatous histology, have primary tumors of the nasopharynx or tumors of unknown origin, and receive systemic treatment after BMM. The median survival times were 61 days (95% CI 41–81) and 70 days (95% CI 54–86) in the derivation and validation cohorts, respectively.

**TABLE 1:**
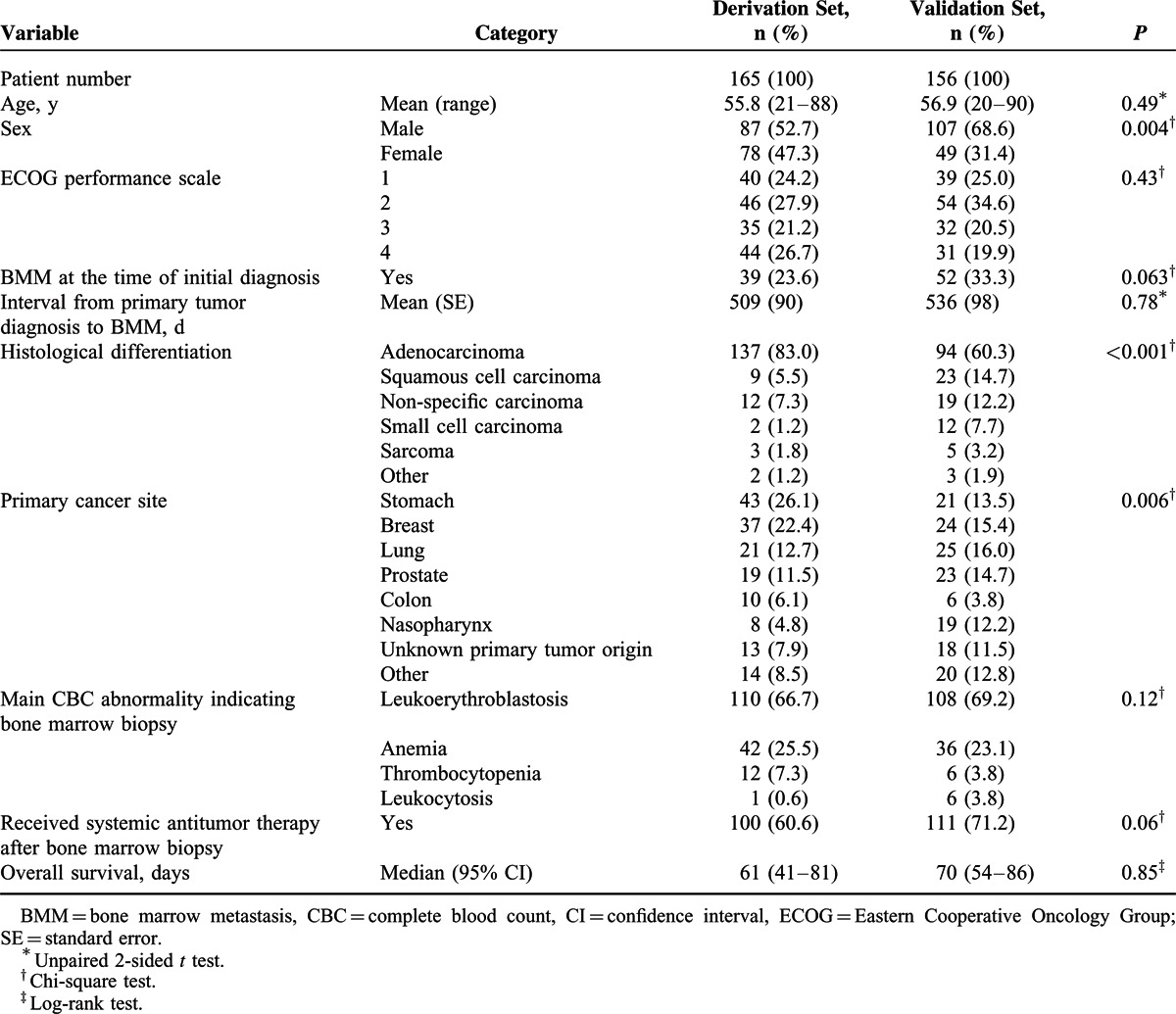
Basic Patient Demographic Data

### Independent Predictive Factors in the Derivation Cohort

Based on a univariate analysis of the data, 7 of the 15 preselected variables had statistically significant effects on survival (Supplementary appendix, http://links.lww.com/MD/A302). However, a multivariate analysis identified ECOG performance scale, primary tumor site, platelet count, and NLR as the only independent prognostic factors.

### Risk Model, Prognostic Group Classification, and Model Accuracy in the Derivation Cohort

The risk model and scoring system of the MMPS generated from the β-coefficients in the multivariate analysis based on the derivation cohort are shown in Table 2. The total MMPS ranged from 0 to 11.5. The patients were stratified into good (total score of 0–4), intermediate (4.5–7.5), and poor (8–11.5) prognostic groups according to the MMPS. Based on the MMPS, 34.5% of the patients were assigned to the good, 35.8% to the intermediate, and 29.7% to the poor prognostic groups.

**TABLE 2:**
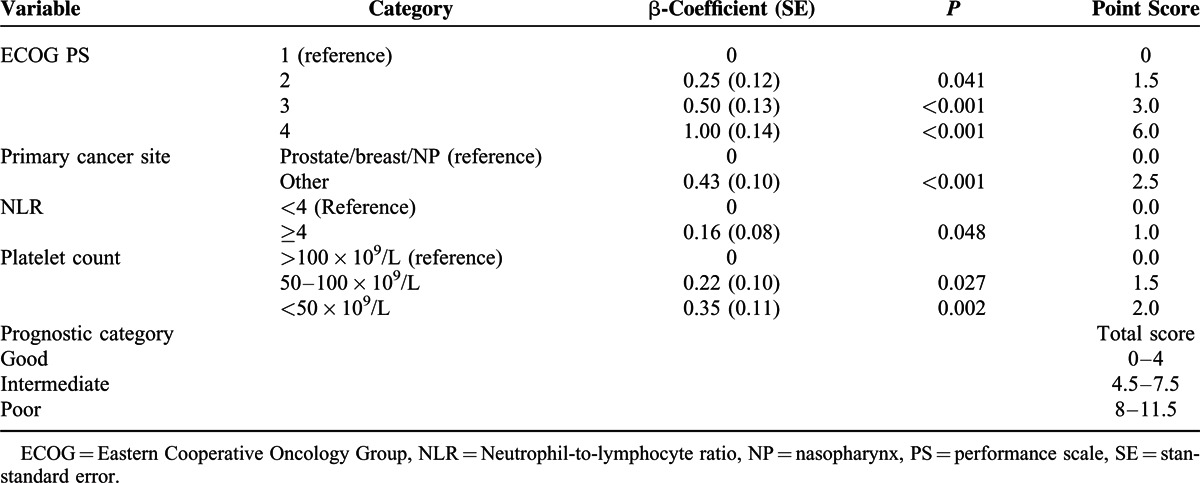
Risk Models and Prognostic Scores for the Derivation Set (n = 165)

**FIGURE 2:**
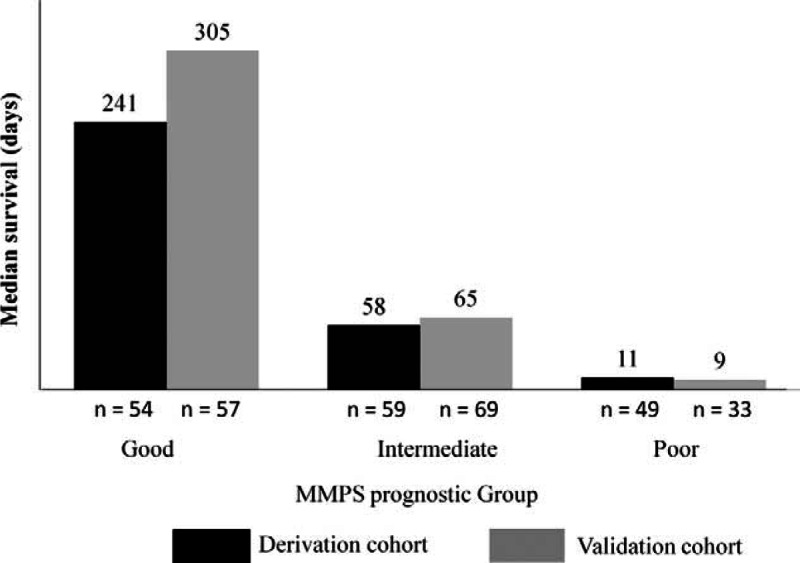
Median survival for patients in the derivation and validation cohorts stratified by the marrow metastasis prognostic score (MMPS) prognostic groups. The number above each column represents the median survival (days) for each prognostic group.

The median survival times of the patients in the different prognostic groups in the derivation cohort are shown in Figure 2. The median survival in the good, intermediate, and poor prognostic groups of the derivation cohort was 241 days (95% CI 152–330, n = 57), 58 days (95% CI 43–73, n = 59), and 11 days (95% CI 4–18, n = 49), respectively (*P* < 0.001 for a comparison between the intermediate or poor groups with the good prognostic group).

**FIGURE 3:**
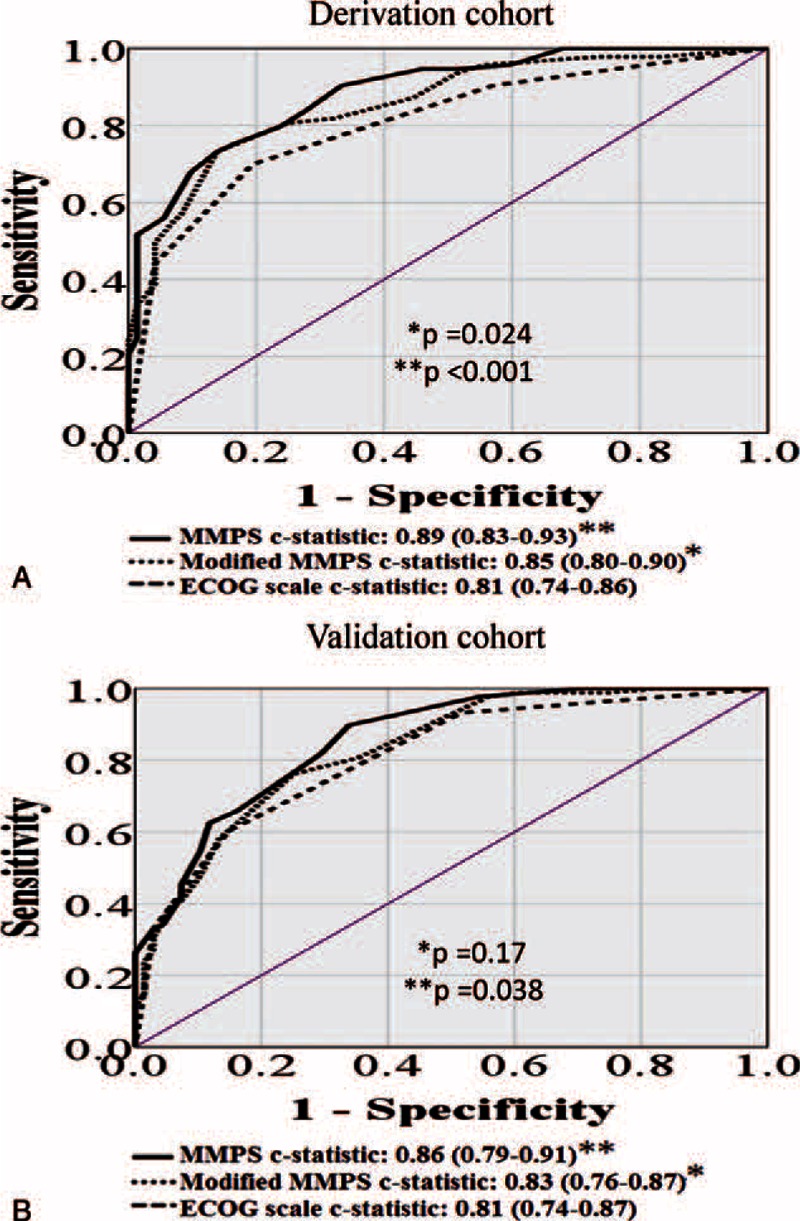
Receiver operating characteristic (ROC) analysis using the marrow metastasis prognostic score (MMPS), modified MMPS (eliminated the tumor site from MMPS) and Eastern Cooperative Oncology Group (ECOG) scale for the outcome of mortality in the derivation cohort at 3 months (A) and in the validation cohort at 3 months (B).

The ROC analysis of mortality at 3, 6, and 12 months using the MMPS resulted in significantly higher c-statistic values than the ECOG scale alone. The c-statistic at 3 months was 0.89 (95% CI 0.83–0.93) for the MMPS compared with 0.81 (95% CI 0.74–0.86) for the ECOG scale (*P* < 0.001). At 6 months, the c-statistic for the MMPS was 0.89 (95% CI 0.84–0.94) compared with 0.82 (95% CI 0.76–0.89) for the ECOG scale (*P* = 0.003). At 12 months, the c-statistic for the MMPS was 0.89 (95% CI 0.83–0.93) compared with 0.82 (95% CI 0.75–0.87) for the ECOG scale (*P* = 0.014). A modified MMPS, which eliminated the tumor site from MMPS, was also calculated of c-statistic value for mortality at 3 months. The c-statistic using the MMPS, modified MMPS and ECOG scale for the prediction of mortality at 3 months is shown in Figures 3A. The c-statistic at 3 months was 0.85 (95% CI 0.80–0.90) for the modified MMPS compared with 0.81 (95% CI 0.74–0.86) for the ECOG scale (*P* = 0.024).

### Validation of the Accuracy of the MMPS

In the validation cohort, the median survival times were 305 (95% CI 216–394, n = 54), 65 (95% CI 51–79, n = 69), and 9 (95% CI 3–14, n = 33) days for patients in the good, intermediate, and poor prognostic groups, respectively, which were comparable to those of the derivation cohort (Figure 2).

The ROC analysis for the validation cohort also resulted in significantly higher c-statistic values for the MMPS than for ECOG scale. At 3 months, the c-statistic for the MMPS was 0.86 (95% CI 0.79–0.91) compared with 0.81 (95% CI 0.74–0.87) for the ECOG scale (*P* = 0.038). At 6 months, the c-statistic for the MMPS was 0.87 (95% CI 0.81–0.92) compared with 0.80 (95% CI 0.73–0.87) for the ECOG scale (*P* = 0.009). Finally, at 12 months, the c-statistic for the MMPS was 0.88 (95% CI 0.82–0.93) compared with 0.77 (95% CI 0.70–0.83) for the ECOG scale (*P* < 0.001). The c-statistic using the MMPS, modified MMPS and ECOG scale to predict mortality at 3 months are shown in Figure 3B. The c-statistic at 3 months was 0.83 (95% CI 0.76–0.87) for the modified MMPS compared with 0.81 (95% CI 0.74–0.86) for the ECOG scale (*P* = 0.17).

### Survival Curves According to the MMPS Categories in the Derivation and Validation Cohorts

**FIGURE 4:**
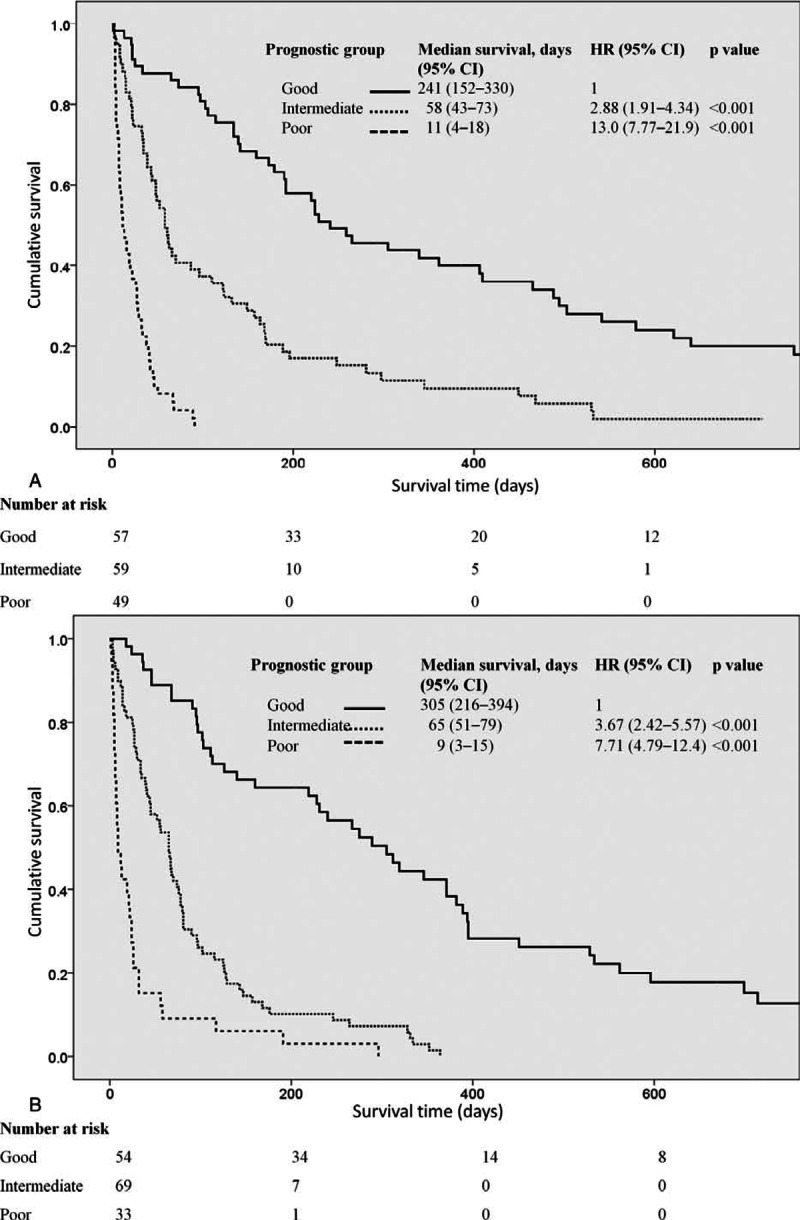
Kaplan-Meier survival curves for cancer patients in the derivation (A) and validation (B) cohorts stratified according to the marrow metastasis prognostic score (MMPS) prognostic group.

On the date that the study was censored, 159 (96.4%) of patients in the derivation cohort and 151 (96.8%) of patients in the validation cohort were deceased. The Kaplan-Meier survival curves for the patients according to the risk model categories in the derivation and validation cohorts are shown in Figure 4A and B. The hazard ratios were significantly different when the poor and intermediate prognostic groups, based on the MMPS, were compared with the good prognostic group in the derivation and validation cohorts (*P* < 0.001 for all groups).

### Subgroup Analysis for Survival Based on Antitumor Therapy in the Derivation and Validation Cohorts

Subgroup analysis for survival based on antitumor therapy within patients categorized by primary tumor site, ECOG scale and MMPS prognostic group was showed in Table 3. In general, patients received antitumor therapies had better survival than those without. Patients with chemotherapy-sensitive tumor types, better ECOG scale and in good MMPS prognostic group were more likely to receive antitumor therapies. Moreover, patients received antitumor therapies also had lower MMPS than those without, independent of their primary tumor site, ECOG scale and MMPS prognostic group.

**TABLE 3:**
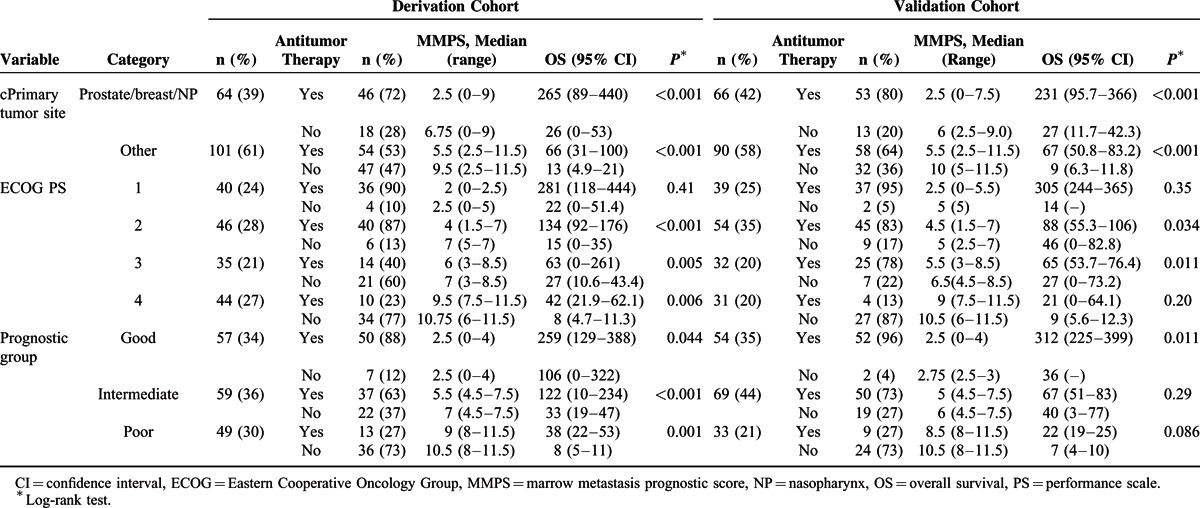
Subgroup Analysis for Survival Based on Antitumor Therapy Within Patients Categorized by Primary Tumor Site, ECOG Scale and MMPS Prognostic Group

## DISCUSSION

In this study, we identified 4 clinical variables that were independent predictors of BMM in patients with solid cancers. These variables were used to generate a simple prognostic score (MMPS) that allows providers to classify patients into 3 distinct categories that correspond to survival. The MMPS was then validated in an independent cohort of patients from 3 other hospitals. The MMPS showed good discrimination and prognostic prediction accuracy in the validation cohort.

The independent predictors of survival time were ECOG scale, primary tumor site, NLR, and platelet count. Most of the prognostic factors identified in this analysis closely resembled those of previously published reports.^1,5–8^ The ECOG scale contributed the most in this model because it represents the general health of the patient and quantifies the activities of daily life. The ECOG performance scale was originally designed to determine whether a patient was eligible to receive chemotherapy and is now widely used to determine eligibility for clinical trials, measure quality of life, predict treatment-related toxicities, and estimate prognosis in oncology practices.^19,20^ Our study showed that the MMPS combining the ECOG scale and other prognostic factors could provide a better risk stratification of survival times than the ECOG scale alone. In addition, subgroup analysis in our study showed patients received antitumor therapy had better outcome than those without antitumor therapy. Patients with bone marrow metastases and good ECOG scale, they will almost undoubtedly have antitumor therapy. For those with borderline ECOG scale (2 or 3), there may be certain concerns regarding a patient's tolerance to the antitumor treatment. In all 4 ECOG scale categories, patients had antitumor therapies showed lower MMPS than those without in both derivation and validation cohorts. We believe that MMPS which combing ECOG scale and other prognostic factor, may helpful to clinician and patients for decision making of antitumor therapy.

Consistent with previous reports, patients with carcinomas that originated in the breast or prostate had better outcomes than did patients with tumors that originated in other sites.^1,6^ In addition, we found that patients with cancer of the nasopharynx, which is prevalent in individuals of East Asian descent, had similar outcomes to those of patients with breast or prostate cancer. Slow-growing tumors and favorable responses to chemotherapy may explain the improved outcomes among patients with these carcinomas. However, this theory cannot explain the dismal outcomes in patients with colorectal cancer and BMM. In contrast to prostate, breast, and nasopharyngeal cancers, colorectal cancer with BMM was less frequently reported. The tumors from colorectal cancer patients with BMM may have behaved distinctly and had different therapeutic responses compared with the tumors of patients that did not have BMM. The MMPS score were constructed to predict outcome in all solid-cancer patients with bone marrow metastases. Concerning the utility of MMPS score in patients with specific appointed cancer type, we eliminated the tumor site from MMPS to create a new model (modified MMPS). The ROC analysis of mortality at 3 months using modified MMPS resulted in significantly higher c-statistic value than ECOG scale alone in derivation cohort. Using MMPS, with or without primary tumor site, showed a significantly better outcome prediction in our model than using ECOG scale alone. Therefore, our model provided valuable prognostic information in patients of both specific and all cancer types.

We previously reported that platelet count was a prognostic factor in patients with BMM.^6^ This study expanded the role of the platelet count in these patient groups. Thrombocytopenia may impact patient outcomes by increasing the incidence of bleeding or thromboembolism sequelae and decreasing the tolerance of subsequent antitumor therapy. In contrast to anemia, thrombocytopenia was more difficult to treat with platelet transfusion because of the short life span of circulating platelets. Therefore, platelet count might be a more reliable marker of bone marrow reserve than hemoglobin in patients with BMM. This could explain why platelet count, but not hemoglobin, was an independent predictor in our study.

The NLR is an emerging inflammatory marker that has been shown to be an independent prognostic factor in numerous epidemiological studies of various cancers.^21^ Higher NLRs have been consistently associated with advanced-stage and aggressive tumors.^22^ The underlying mechanism may involve negative regulation of tumor progression by lymphocytopenia through the induction of cytotoxic cell death and inhibition of tumor proliferation.^23,24^ In contrast, an elevated neutrophil count may decrease the cytolytic activities of lymphocytes or natural killer cells and could promote tumor growth.^25,26^ The prognostic value of the NLR that we report in this study is novel. In comparison to other systemic inflammatory markers such as C-reactive protein^27^ and the Glasgow Prognostic Score,^28^ the NLR is easily calculated from a routine CBC with a differential, an essential blood test and a main reason for a subsequent bone marrow biopsy in cancer patients when BMM is suspected.

Previous studies have reported that antitumor therapy following BMM is a strong positive prognostic factor. However, we did not include this variable in our survival analysis because it could be confounded by other variables such as performance scale, type of primary tumor, or previous treatment response before the diagnosis of BMM. In order to make treatment decisions regarding antitumor therapy for patients that presented with BMM initially, the primary tumor had to be identified. Therefore, this variable could not be used immediately after BMM was confirmed. From a clinical perspective, the ability to predict patient outcome immediately after BMM diagnosis may provide valuable prognostic information to the clinician, the patient, and the family. To preserve the utility of the model for decision making, we only used clinical variables that are easily accessible at the time of diagnosis with BMM. The specific cancer type, type of treatment, and post-treatment variables were not included in the analysis, although these factors could impact mortality. Therefore, this model could be used in routine clinical practice to predict the outcomes of all solid-cancer patients diagnosed with BMM.

We noted several statistical issues regarding the derivation and validation of the demographic data. The validation cohort contained more male patients, had a higher proportion of patients with tumors of the nasopharynx or of unknown origin, and had a higher proportion of patients that presented with BMM initially. This may reflect the higher proportion of patients with non-adenocarcinomatous histology, metastasis limited to the bone marrow and bone, and the use of antitumor therapy after BMM. Although significant differences in these variables existed between the 2 cohorts, they were not independent prognostic factors that contributed to the MMPS (except for tumor site), and hence, the statistical difference in the demographic data did not influence the accuracy of the prognostic prediction.

We believe that the MMPS will assist patients and clinicians with making treatment decisions for advanced-stage cancer with BMM. Patients with a good prognosis may be encouraged to receive more aggressive antitumor therapy. In contrast, a bone marrow biopsy may be futile in patients with a poor prognosis, given that the median survival of these patients was only 11 and 9 days in our derivation and validation cohorts, respectively. Appropriate end-of-life care should be provided for these patients.

The strengths of this study are that the MMPS was developed using a large sample size from the largest medical center in Taiwan and was externally validated using a large number of samples from multiple hospitals. This is the largest study to evaluate prognostic factors in adults with solid tumors and BMM. The clinical variables of the MMPS are accessible and are available at the time of diagnosis. Therefore, this model is widely applicable and clinically relevant. However, there are limitations. First, this was a retrospective study with a long recruitment period, during which practice patterns might have changed. Second, because only patients that were symptomatic with BMM were included, the risk model may not be generalized to patients with bone marrow micrometastases or clinically suspicious patients who did not undergo a bone marrow biopsy. Third, our study included a variety of cancer types and various antitumor therapies. The effectiveness of each antitumor therapy on a particular cancer may potentially affect survival. Last and most importantly, our analysis included only patients with BMM confirmed by bone marrow examination; as such there was selection bias regarding which patients were offered bone marrow study. Because of the rarity of the disease, a well-designed multisite prospective study is necessary to address these limitations.

In conclusion, we developed and validated a risk model that accurately predicted survival in adult patients with solid cancers and BMM. This scoring system may be used to facilitate treatment decisions.
