# Development and Validation of a Prognostic Score to Predict Survival in Adult Patients With Solid Tumors and Bone Marrow Metastases: Erratum

**DOI:** 10.1097/01.md.0000469401.76367.85

**Published:** 2015-07-02

In the article “Development and Validation of a Prognostic Score to Predict Survival in Adult Patients With Solid Tumors and Bone Marrow Metastases”,^1^ which appeared in Volume 94, Issue 23 of *Medicine*, Dr. Shau-Hsuan Li's name was inadvertently omitted from the byline. Additionally, the corresponding author for this article should be Chang-Hsien Lu, not Ki Deok Park. The article has since been corrected online.
